# Preclinical Evaluation and Dosimetry of [^111^In]CHX-DTPA-scFv78-Fc Targeting Endosialin/Tumor Endothelial Marker 1 (TEM1)

**DOI:** 10.1007/s11307-020-01479-8

**Published:** 2020-01-28

**Authors:** Francesco Cicone, Thibaut Denoël, Silvano Gnesin, Nicolo Riggi, Melita Irving, Gopinadh Jakka, Niklaus Schaefer, David Viertl, George Coukos, John O. Prior

**Affiliations:** 1 Department of Nuclear Medicine and Molecular Imaging, Lausanne University Hospital and University of Lausanne, Lausanne, Switzerland; 2 Department of Experimental and Clinical Medicine, Unit of Nuclear Medicine, “Magna Graecia” University of Catanzaro, Catanzaro, Italy; 3 Institute of Radiation Physics, Lausanne University Hospital and University of Lausanne, Lausanne, Switzerland; 4 Experimental Pathology Service, Institute of Pathology, Lausanne University Hospital and University of Lausanne, Lausanne, Switzerland; 5 Department of Oncology, Ludwig Institute for Cancer Research, Lausanne Branch, University of Lausanne, CH-1066 Epalinges, Switzerland

**Keywords:** TEM1, Internal dosimetry, Radiolabeled scFv78-Fc, Endosialin, CD248, Biodistribution, Ewing’s sarcoma, Neuroblastoma, Molecular imaging, Dose extrapolation

## Abstract

**Purpose:**

Endosialin/tumor endothelial marker-1 (TEM1) is an attractive theranostic target expressed by the microenvironment of a wide range of tumors, as well as by sarcoma and neuroblastoma cells. We report on the radiolabeling and preclinical evaluation of the scFv78-Fc, a fully human TEM1-targeting antibody fragment cross-reactive with mouse TEM1.

**Procedures:**

The scFv78-Fc was conjugated with the chelator *p*-SCN-Bn-CHX-A”-DTPA, followed by labeling with indium-111. The number of chelators per molecule was estimated by mass spectrometry. A conventional saturation assay, extrapolated to infinite antigen concentration, was used to determine the immunoreactive fraction of the radioimmunoconjugate. The radiopharmaceutical biodistribution was assessed in immunodeficient mice grafted with Ewing’s sarcoma RD-ES and neuroblastoma SK-N-AS human TEM1-positive tumors. The full biodistribution studies were preceded by a dose-escalation experiment based on the simultaneous administration of the radiopharmaceutical with increasing amounts of unlabeled scFv78-Fc. Radiation dosimetry extrapolations to human adults were obtained from mouse biodistribution data according to established methodologies and additional assumptions concerning the impact of the tumor antigenic sink in the cross-species translation.

**Results:**

[^111^In]CHX-DTPA-scFv78-Fc was obtained with a radiochemical purity > 98 % after 1 h incubation at 42 °C and ultrafiltration. It showed good stability in human serum and > 70 % immunoreactive fraction. Biodistribution data acquired in tumor-bearing mice confirmed fast blood clearance and specific tumor targeting in both xenograft models. The radiopharmaceutical off-target uptake was predominantly abdominal. After a theoretical injection of [^111^In]CHX-DTPA-scFv78-Fc to the reference person, the organs receiving the highest absorbed dose would be the spleen (0.876 mGy/MBq), the liver (0.570 mGy/MBq) and the kidneys (0.298 mGy/MBq). The total body dose and the effective dose would be 0.058 mGy/MBq and 0.116 mSv/MBq, respectively.

**Conclusions:**

[^111^In]CHX-DTPA-scFv78-Fc binds specifically to endosialin/TEM1 *in vitro* and *in vivo*. Dosimetry estimates are in the range of other monoclonal antibodies radiolabeled with indium-111. [^111^In]CHX-DTPA-scFv78-Fc could be potentially translated into clinic.

**Electronic supplementary material:**

The online version of this article (10.1007/s11307-020-01479-8) contains supplementary material, which is available to authorized users.

## Introduction

Endosialin (CD248), also known as tumor endothelial marker-1 (TEM-1), is a C-type lectin-like transmembrane glycoprotein of about 165 kDa, which was initially identified as an antigen of human fetal fibroblasts and thought to be associated with tumor vascular endothelium [1–3]. Later studies have shown that, during mouse ontogenesis, endosialin is preferentially expressed by mesenchymal stromal fibroblasts and by pericytes, being progressively lost with further development [4]. In human adults, the cellular expression of endosialin appears to be restricted to few normal tissues, including endometrial stroma, smooth muscle of the colon and prostate [5, 6]. Other studies have suggested a role for endosialin in the expansion of secondary lymphoid organs following infections or immunogenic triggers [7–9]. In most solid cancers, endosialin is overexpressed by tumor-associated stromal fibroblasts and perivascular pericytes, but it is absent on tumor endothelium [10, 11]. Furthermore, a higher degree of endosialin expression seems to correlate with higher tumor aggressiveness and worse survival outcomes [12–14].

The extracellular domain of endosialin binds to multiple matrix proteins, including fibronectin, collagen I and IV and the Mac-2 BP/90 K protein. These interactions translate into intracytoplasmic downstream signals, which contribute to stromal cells activation and increased proteases activity, eventually leading to tumor cells proliferation, adhesion and migration [15–17]. In addition, it has been shown that the presence of endosialin is essential for the cellular growth and the migration of pericytes, which are key regulators of tumor neoangiogenesis [18, 19]. Interestingly, in some tumors, namely sarcoma and neuroblastoma, the expression of endosialin/TEM1 is not only limited to the tumor stroma and neovasculature but it is also present on tumor cell surface [11, 20–22].

Owing to this favorable expression pattern and fundamental interplays within the tumor microenvironment, endosialin has been identified as an attractive target for tumor therapy and molecular imaging, and several endosialin-targeting antibodies have been developed for oncological applications [23].

The MORAb-004 (ontuxizumab, Morphotek), a humanized full IgG antibody, is in Phase 1/2 clinical trials for the therapy of several neoplasms [24–27] and has been previously labeled with two positron-emitting radionuclides, iodine-124 [28] and zirconium-89 [29], respectively. Another humanized monoclonal antibody, the hMP-E-8.3, has been conjugated with a duocarmycin derivative, showing significant antitumor activity in an osteosarcoma mouse model [30].

A fully human antibody scFv fragment, cross-reactive with mouse endosialin/TEM1, has also been developed as a vector for optical probes and immunotoxin-based therapy [31, 32]. The scFv78, a derivative of this scFv fragment, was fused to an immunoglobulin Fc (scFv78-Fc) in order to obtain a bivalent molecule with improved pharmacokinetic and targeting properties [33]. In the present paper, we report on the preclinical evaluation of the scFv78-Fc labeled with the single-photon emitting radionuclide indium-111. The radioimmunoconjugate was tested both *in vitro* and *in vivo*, and radiopharmaceutical biodistribution data were used for radiation dose extrapolations to human adults. Small animal imaging and dosimetry of the scFv78-Fc labeled with the unconventional positron-emitting radionuclide terbium-152 were recently reported elsewhere [34]. This previous publication included also part of the data presented here in full, namely the biodistribution experiment in Ewing’s sarcoma RD-ES tumor-bearing mice [34].

## Methods

### Conjugation of scFv78-Fc with *p*-SCN-Bn-CHX-A”-DTPA and Radiolabeling with Indium-111

The scFv78-Fc fusion protein targeting endosialin/TEM1 was obtained as previously described [33]. A solution of scFv78-Fc (1.19 mg/ml, 960 μl) was concentrated to *ca.* 200 μl by ultrafiltration (Amicon Ultra, 0.5 ml, 50 kD) in two successive 450 μl additions and spins (2 × 4 min at 9000 g). Subsequently, 67 μl (10 eq.) of a freshly made solution of 1.0 mg of *p*-SCN-Bn-CHX-A*”*-DTPA · 3 HCl (Macrocyclics, Cat-N° B-355) in dimethyl sulfoxide (DMSO) (50 μl) and carbonate-bicarbonate (CBC) buffer (0.2 M, pH 9.1, 1 ml) were added to the concentrated scFv78-Fc solution and incubated for 1 h at 42 °C. Then, dilution with 500 μl of NaCl 0.9 % was effected, and the excess chelator was removed by ultrafiltration (3 × 6 min at 9000 g). The *ca.* 40 μl solution was diluted with 190 μl of NaCl 0.9 % to give a final concentration of 5 mg/ml CHX-DTPA-scFv78-Fc.

**Fig. 1:**
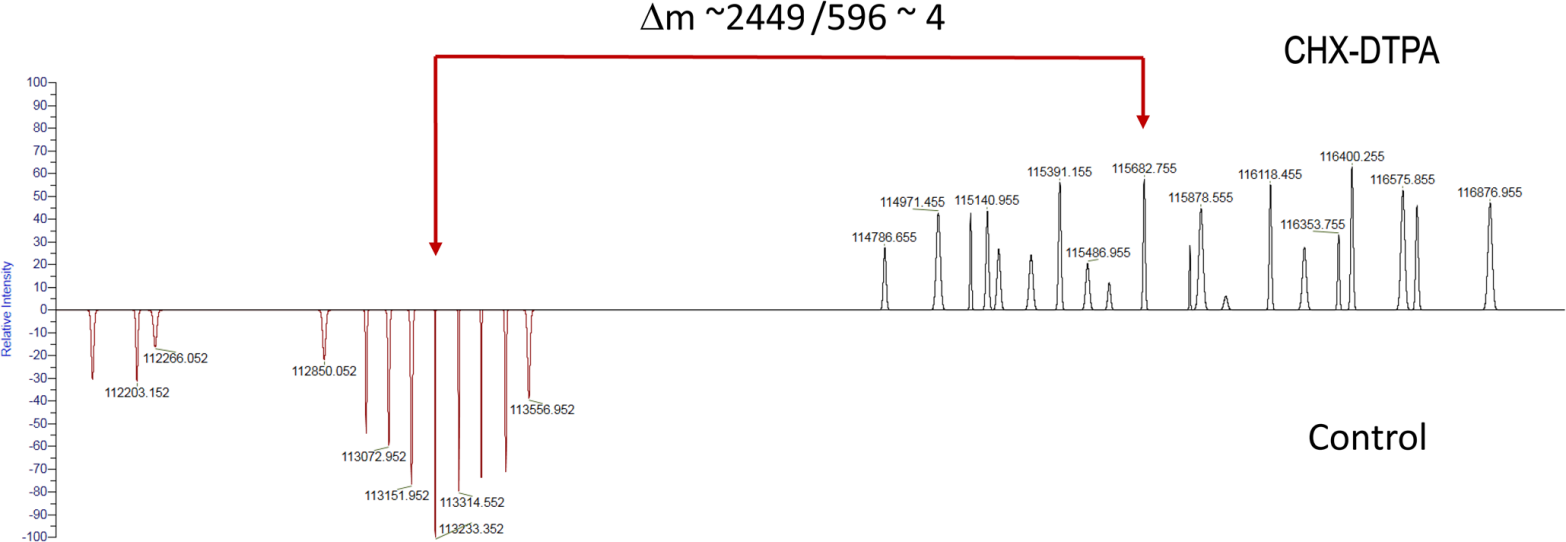
Mass spectrometry of scFv78-Fc (bottom) and of CHX-DTPA-scFv78-Fc (top). The delta of m/z 2449 between mean peaks suggests an average of 4 CHX-DTPA per antibody with a distribution between 3 and 6.

Mass spectrometry (MS) was performed using a Q Exactive HF (Thermo Fisher) operating in the high mass range in order to quantify the mean number of chelators per antibody. Samples were diluted to 1 μg/μl in NH_4_OAc (pH 7, 50 mM) and injected in a MAbPac™ SEC-1 column (Thermo Fisher Scientific) using NH_4_OAc (pH 7, 50 mM) at 0.3 ml/min as mobile phase. MS spectra were acquired in the 300–8000 *m/z* at a resolution set to 15 K. The MS spectra were deconvoluted using Protein Deconvolution (Thermo Fisher Scientific). The analysis showed a distribution between 3 and 6 CHX-DTPA chelators per scFv78-Fc, with a mean value of about 4 chelators per antibody (Fig. 1).

[^111^In]CHX-DTPA-scFv78-Fc was obtained by adding a commercially available [^111^In]indium chloride solution (200 MBq, Mallinckrodt) to a mixture of NH_4_OAc (pH 5.4, 0.4 M, 100 μl) and CHX-DTPA-scFv78-Fc (50 μl, 250 μg). The radiolabeling yield was assessed by instant thin layer chromatography on silica gel (iTLC-SG) with citrate buffer pH 4.6.

### Serum Stability

Stability was verified by incubating an aliquot of [^111^In]CHX-DTPA-scFv78-Fc (16 MBq, 50 μg) in 1 ml of human serum for 96 h at 37 °C. The solution was centrifuged for 5 min at 10,000 G before spotting 5 μl of solution on an iTLC-SG (citrate buffer pH 4.6). The radiolabeled antibody retained activity at Rf 0, and the unchelated indium-111 migrated at the front.

### Affinity

The binding affinity of CHX-DTPA-scFv78-Fc to human TEM1 antigen was obtained by a single cycle titration method [35] using a Biacore T200 instrument (GE Healthcare Life Sciences), according to procedures similar to those described in [32]. In brief, after capturing biotinylated TEM1 antigen on a streptavidin-coated sensor chip (GE Healthcare Life Sciences), three fold serial dilutions of CHX-DTPA-scFv78-Fc (1–81 nM) were injected at a flow rate of 30 μl/min and a contact time of 2 min. After 5 min of dissociation, the TEM1 surface was regenerated using 2 M NaCl. Binding signals were processed with Biacore T200 Evaluation Software by subtracting the responses of an empty streptavidin surface and of a series of buffer blanks. Kinetic analysis was performed over a concentration series of 1–8 nM using a 1:1 binding model including a mass transport parameter in Biacore T200 Evaluation Software to fit association rate (*k*_a_) and dissociation rate (*k*_d_) constants and calculate the binding affinity (*K*_D_ = *k*_d_/*k*_a_).

### Measurement of Immunoreactivity

The immunoreactive fraction (*r*) of [^111^In]CHX-DTPA-scFv78-Fc was assessed by a conventional saturation assay, extrapolated to infinite antigen by a rectangular hyperbola. A high-binding 96-well microplate (Costar #3590, Corning) was coated in triplicate with decreasing amount of recombinant human TEM1 protein in phosphate buffered saline (PBS) (100 μL, 0.125–8 μg/ml in 2-fold serial dilutions) for 16 h at 4 °C. After flicking the plate and washing three times with PBS (200 μl), the wells were blocked for 2 h with blocking buffer (2 % BSA, 1 μM EDTA and 0.05 % Tween-20 in PBS). Three empty wells were also blocked for nonspecific binding determination. The wells were rinsed with PBS before the addition of a solution of [^111^In]CHX-DTPA-scFv78-Fc in blocking buffer (100 μL, 0.025 μg/ml). The same volume of [^111^In]CHX-DTPA-scFv78-Fc solution was also pipetted in (i) three γ-counter tubes as an internal control for the measurement of the total activity and (ii) the three empty wells that were previously blocked for a blank triplicate (*U*_*Blank*_). The plate was incubated for 16 h at 4 °C. The supernatant was pipetted off in γ-counter tubes, the wells rinsed once on ice with cold PBS and the pooled liquid was counted in a calibrated gamma-counter (Wallac Wizard, Perkin Elmer). The specific binding (*B*_Spec_) was calculated for each dilution step as the difference in counts between the mean unbound supernatant of the blank and the unbound supernatant (*U*): ${B}_{Spec}={\overline{U}}_{Blank}-U$. A hyperbolic fit was applied using Graphpad Prism to provide *r* from the extrapolation of $\frac{B}{T}=\frac{B_{Spec}}{{\overline{U}}_{Blank}}$ at infinite antigen concentration. The assumption was made of a single binding site per antigen. The mathematical aspect of this method was fully validated in a separate publication [36].

### Tumor Cell Lines and Tumor Models

Two endosialin/TEM1-positive human tumor cell lines, the Ewing’s sarcoma RD-ES and the Neuroblastoma SK-N-AS, and one endosialin/TEM1-negative cell line, the fibrosarcoma HT-1080, were chosen for the experiments based on previously published papers using the commercially available MORAb-004 [29, 37]. RD-ES cells were purchased from DSMZ, Germany (n° ACC 260) and cultured in RPMI-1640 medium, 15 % fetal bovine serum (FBS) and 1 % penicillin/streptomycin solution. SK-N-AS cells (ATCC CRL-2137) were cultured in DMEM, 10 % FBS, 5 % non-essential amino acids and 1 % penicillin/streptomycin solution. HT-1080 cells (ATCC® CCL-121™) were cultured in EMEM medium, 10 % FBS and 1 % penicillin/streptomycin solution.

Tumor cells were injected subcutaneously in 8–12 weeks old female common gamma KO Balb/c mice (3 × 10^6^ RD-ES, 1 × 10^6^ SK-N-AS and 3 × 10^6^ HT-1080 cells injected, respectively) and allowed to grow for 3 weeks approximately. Mice were used for the experiments when maximum tumor diameter was about 10 mm. Attention was paid to make mice groups homogeneous in terms of tumor diameters, in order to avoid the variability that might be introduced by the presence of necrotic areas in largest tumors.

All applicable institutional and/or national guidelines for the care and use of animals were followed. In particular, all animal experiments in the present study were conducted according to the Swiss federal law on animal experimentation under the authorization number VD-2993.

### Characterization of Tumor Models: Flow Cytometry

The scFv78-Fc was used to stain RD-ES, SK-N-AS and HT-1080 cell lines for endosialin expression by flow cytometry (FACS). Tumor cells (0.5 × 10^6^) were treated in 200 μL PBS/FBS 2 % buffer with scFv78-Fc (1 μg/ml) in a tube and incubated at 4 °C for 1 h. After washing, 50 μL of an APC-conjugated anti-human-Fc antibody were added and incubated for 30 min at 4 °C, protected from light. Cells were washed and resuspended in 200 μL FACS buffer for analysis. An isotype antibody, the APC-conjugated antibody and unstained cells were used as negative controls.

### Characterization of Tumor Models: Immunohistochemistry

Paraffin-embedded sections of RD-ES and SK-N-AS xenograft tumors were stained with a purified mouse IgG_K_ anti-endosialin monoclonal antibody (Clone B1/35, cat# MAB2626, Merck Millipore), cross-reactive with human (1:1000 dilution; Millipore, Chemicon). Horseradish peroxidase staining was performed using biotin-conjugated rabbit anti-mouse Ig (Vector Laboratories) and was revealed with a DAKO DAB Kit (Dako).

### Biodistribution Experiments

A preliminary antibody dose-escalation study was undertaken in order to assess the non-tumor tissue antigenic “sink effect” and to optimize the biodistribution of the radioimmunoconjugate [38]. In particular, a saline solution containing 0.2 μg of [^111^In]CHX-DTPA-scFv78-Fc was injected alone or co-injected with increasing amounts of unlabeled scFv78-Fc in RD-ES tumor-bearing mice (average injected activity: 150 kBq). Six groups of mice received 0.2 μg (*n* = 2 mice), 5 μg (*n* = 2 mice), 25 μg (*n* = 5 mice), 50 μg (*n* = 5 mice), 200 μg (*n* = 4 mice) and 1 mg (*n* = 2 mice) of total antibody injected, respectively. Two HT-1080 tumor-bearing mice were injected with 180 kBq [^111^In]CHX-DTPA-scFv78-Fc (25 μg of total injected antibody) and used as negative controls. Mice were bled by cardiac puncture and sacrificed by cervical dislocation under anesthesia with isoflurane (2 % in 1 L/min medical air), 24 h after injection. Organs were harvested and weighed, and radioactivity was counted on a calibrated gamma-counter. Tissue uptake was expressed as % of injected activity/g of tissue (%IA/g). All measurements were corrected for possible radiopharmaceutical extravasations by subtracting the radioactivity counted in the tail from the total injected activity. No decay correction was applied.

The choice of the amount of total antibody to inject in order to optimize the radiopharmaceutical biodistribution was based on the uptake in the tumors and in the most relevant organs, as well as on the tumor-to-organ ratios. The Kruskal–Wallis test was used to compare uptake values between the six groups of mice stratified according to the different amounts of total antibody injected. The Mann–Whitney *U* test was used to compare uptake values between the two groups of mice showing the most favorable biodistribution profiles. Probability values of less than 0.05 were considered significant. Statistical analysis was performed using GraphPad Prism 8.

For full biodistribution experiments, RD-ES and SK-N-AS tumor-bearing mice were injected intravenously with 150 kBq (range: 30–440 kBq) [^111^In]CHX-DTPA-scFv78-Fc (25 μg of total injected antibody, corresponding to 1 mg/Kg) and sacrificed 4, 24, 48 and 96 h after injection (3–5 mice per group).

### Human Dosimetry Extrapolations

Human radiation dose estimates of [^111^In]CHX-DTPA-scFv78-Fc were based on the biodistribution results on RD-ES-bearing mice, injected with [^111^In]CHX-DTPA-scFv78-Fc (25 μg of total antibody injected) and sacrificed at four different time points (4, 24, 48 and 96 h post injection). For each time point, the activity in each source organ of each single animal was normalized by the total injected activity to obtain the normalized injected activity (nA). For each source organ at each time point, an averaged nA ± 1 standard deviation (SD) value across all the animals used for the experiment was obtained. For the rest-of-body, the nA at each time-point was calculated by multiplying the rest-of-body mass (25 g mouse model–sum of the masses of all other considered source organs) by the normalized mass-activity concentration (g^−1^) measured in the muscle, which was taken as the background. Uterus and ovaries were harvested and counted together since a previous biodistribution study on the TEM1-targeting Morab-004 labeled with iodine-124 showed similar tracer kinetics in these organs [28].

A mono-exponential fit extended to infinite beyond the last measured data point was used to derive time-integrated activity coefficients (TIACs) by analytical time-integration of source organ normalized time-activity curves (nTACs) obtained with average nA, nA + SD and nA-SD values, respectively. In the spleen and in the mouse female reproductive organs (*i.e.* uterus and ovaries), however, radioactivity was still in the uptake phase 48 h post injection. Therefore, for these two body districts, between t = 0 and t = 96 h, the TIAC was obtained by trapezoidal integration using Matlab software (Release 2017a, The MathWorks, Inc., Natick, Massachusetts, USA), whereas a mono-exponential analytical integration to infinite was calculated after the last measure, assuming the indium-111 physical decay.

To generate a specific TIAC for uterus and ovaries separately, the total number of disintegrations in these organs was partitioned proportionally to their respective masses reported in the ICRP-89 human female reference phantom [39]. Similarly, we partitioned the total number of disintegrations measured in the mouse colon proportionally to the masses of its components (left, right and rectum) according to the ICRP-89 human reference phantom [39].

It should be noted that only a sample of the total blood can be harvested from cardiac puncture in the living mouse. Therefore, the corresponding TIAC accounts only for a fraction of the actual total blood TIAC. In order to generate a blood TIAC, the following formula was adopted:

$$ \mathrm{TIAC}\left(\mathrm{blood}\right)=\frac{\mathrm{TIAC}\left(\mathrm{blood}\ \mathrm{sample}\right)}{\mathrm{Blood}\ \mathrm{sample}\ \mathrm{mass}\ \left(\mathrm{g}\right)}\times \mathrm{Mouse}\ \mathrm{total}\ \mathrm{blood}\ \mathrm{pool}\ \left(\mathrm{g}\right) $$

The blood sample mass, averaged across all the animals used for the experiment, was 0.672 g. As the mouse total blood pool, we adopted the value of 1.46 g, which considers a total mouse blood volume of 58.5 ml/kg of body weight [40] and a density of 1 g/ml.

Another important aspect is the evaluation of the contribution of the tumor sink. When extrapolating human organ doses from biodistribution obtained in tumor-bearing mouse, it should be considered that the tumor is relatively large compared with the mass of the animal. Consequently, the availability of the antibody for non-tumor tissues would be much higher in absence of such antigenic sink. On the contrary, in humans, the tumors are relatively much smaller than in experimental xenograft mouse models. To take into account this factor, we decided to redistribute the tumor TIAC into mouse source-organ TIACs according to the following formula:

$$ {TIAC}_{m, correct}(organ)={TIAC}_m(organ)+\left[{TIAC}_m(tumor)\times \frac{TIAC_m(organ)}{TIAC_m(WB)-{TIAC}_m(tumor)}\right] $$

Human source-organ TIACs (TIACs_h_ in units of MBq·h/MBq) were extrapolated from murine TIACs (TIACs_m_) according to the Swiss Federal guidelines [41] by applying the formula:

$$ {\mathrm{TIAC}}_h\left(\mathrm{organ}\right)={\mathrm{TIAC}}_m\left(\mathrm{organ}\right)\times \left(\frac{m{\left(\mathrm{organ}\right)}_h}{m{\left(\mathrm{organ}\right)}_m}\right)\times \left(\frac{BW_m}{BW_h}\right) $$

where m(organ)_h_ are the specific organ masses of the adult male and female phantoms, m(organ)_m_ are the averaged organ masses measured across all mice used for the experiment and reported in [34]. BW_m_ is the total-body mass chosen as a reference (*i.e.* 25 g) [42], and BW_h_ are the adult male and female total-body masses according to [39], *i.e.* 73 and 60 kg, respectively.

A specific procedure was dedicated to the extrapolation of the TIAC_h_ for the red marrow (red marrow TIAC_h_) and the heart cavity (heart cavity TIAC_h_) from blood TIAC_h_. The red marrow TIAC_h_ was obtained by multiplying the blood TIAC_h_ by the factor m(red marrow)_h_/m(blood)_h_ for the adult male and female models, respectively. The same activity concentration in the blood and in the red marrow was assumed. Similarly, to obtain the heart cavity TIAC_h,_ the blood TIAC_h_ was multiplied by the factor m(heart cavity)_h_/m(blood)_h_ for the adult male and female models, respectively.

Finally, the TIACs_h_ were entered into the OLINDA/EXM® 2.0 (HERMES Medical Solution AB, Stockholm, Sweden) software kinetic module [43] to derive organ absorbed doses for the adult male, female and reference person, as well as the effective dose for the reference person.

## Results

### Radiolabeling and *in vitro* Assessment of [^111^In]CHX-DTPA-scFv78-Fc

**Fig. 2:**
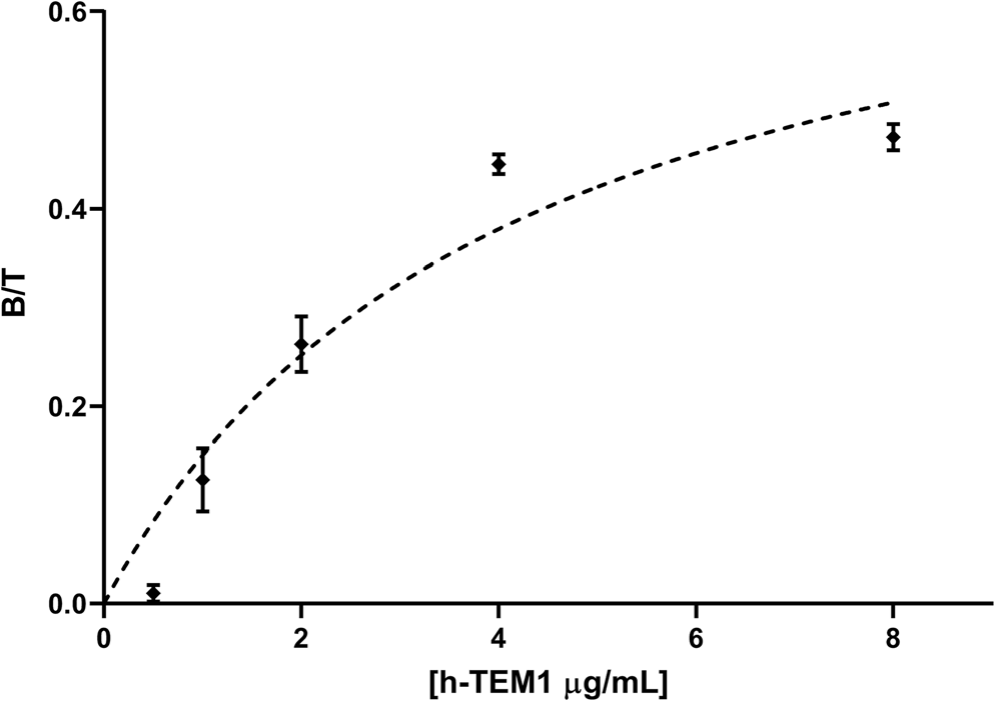
Immunoreactive fraction assay. The immunoreactive fraction *r* was extrapolated by a rectangular hyperbolic fit of experimental values (0.025 μg/ml of radiolabeled antibody). The goodness of fit was estimated by the R^2^ metric (*r* = 77 %, R^2^ = 0.9187).

After 1 h heating at 42 °C, a 80–90 % radiochemical yield of [^111^In]CHX-DTPA-scFv78-Fc was obtained. The final product was diluted with NaCl 0.9 % and purified by ultrafiltration (> 98 % radiochemical purity). The activity at end of synthesis amounts to 9 × 10^16^ Bq/mol of CHX-DTPA-scFv78-Fc. The product was homogeneous (> 97 % iTLC) after 96 h at 37 °C in human serum, indicating good stability. *K*_D_ was 4.5 nM. The immunoreactive fraction *r* extrapolated to infinite antigen excess was *=* 77 % (Fig. 2).

### Characterization of Tumor Models

**Fig. 3:**
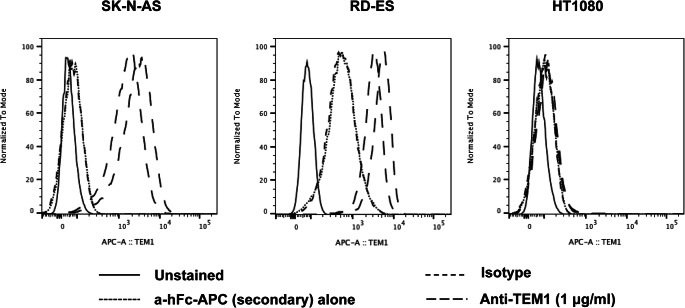
FACS staining for endosialin/TEM1 of cellular tumor models. Both SK-N-AS cells and RD-ES cells confirmed to be positive for TEM1 antigen, with SK-N-AS showing a higher signal compared with the RD-ES cells. In contrast, staining of HT-1080 cells was negative.

**Fig. 4:**
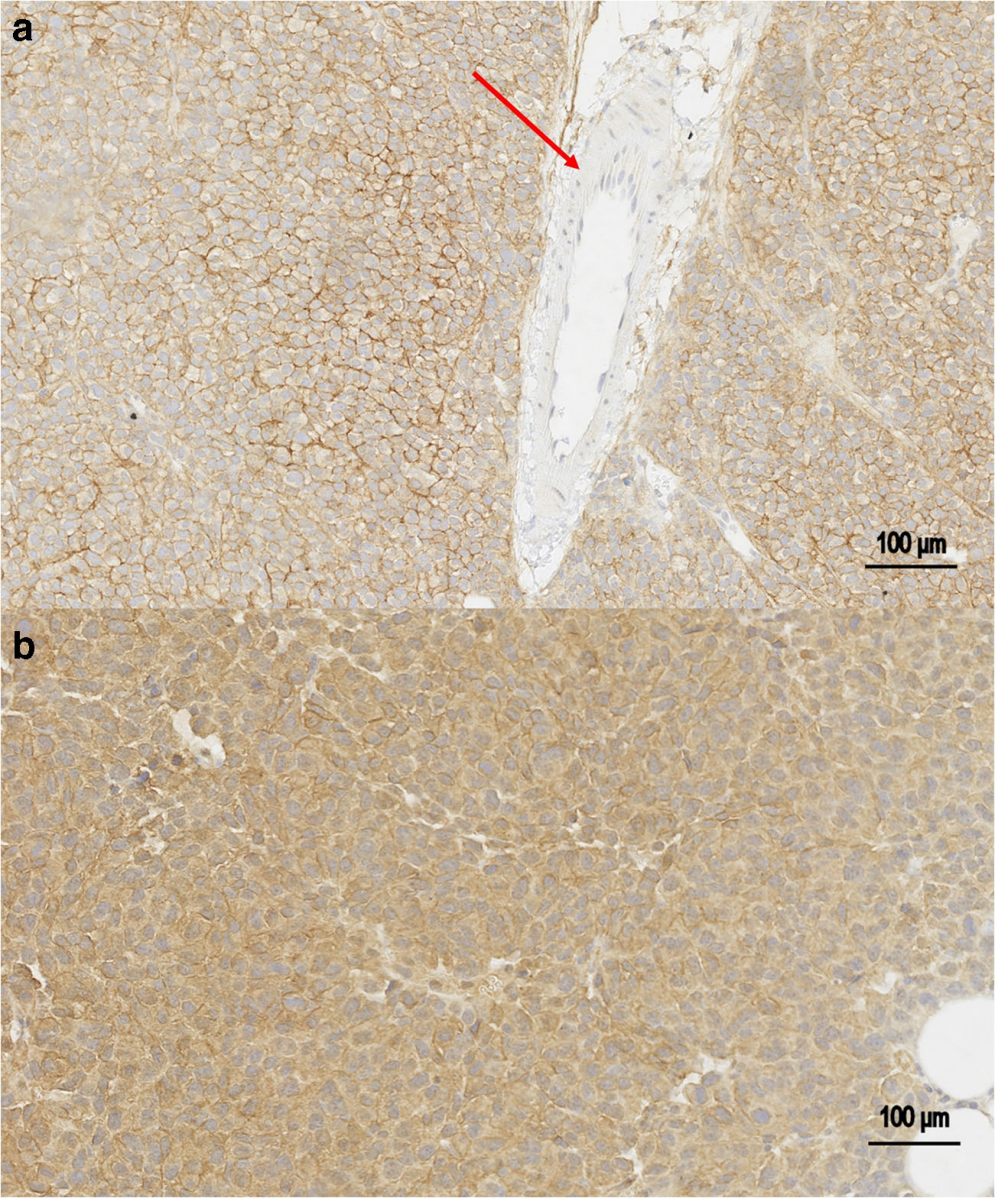
Immunohistochemistry staining for endosialin/TEM1. Sections of RD-ES and SK-N-AS tumors are shown in **a** and **b**, respectively. Results were coherent with those of FACS, showing more intense staining by SK-N-AS than by RD-ES cells. The red arrow indicates absence of endosialin/TEM1 staining by endothelial cells.

FACS (Fig. 3) and immunohistochemistry staining (Fig. 4) confirmed the positivity of both RD-ES and SK-N-AS tumor models for endosialin/TEM1. In contrast, FACS staining of HT-1080 cells for endosialin/TEM1 was negative (Fig. 3).

### Biodistribution Experiments

**Fig. 5:**
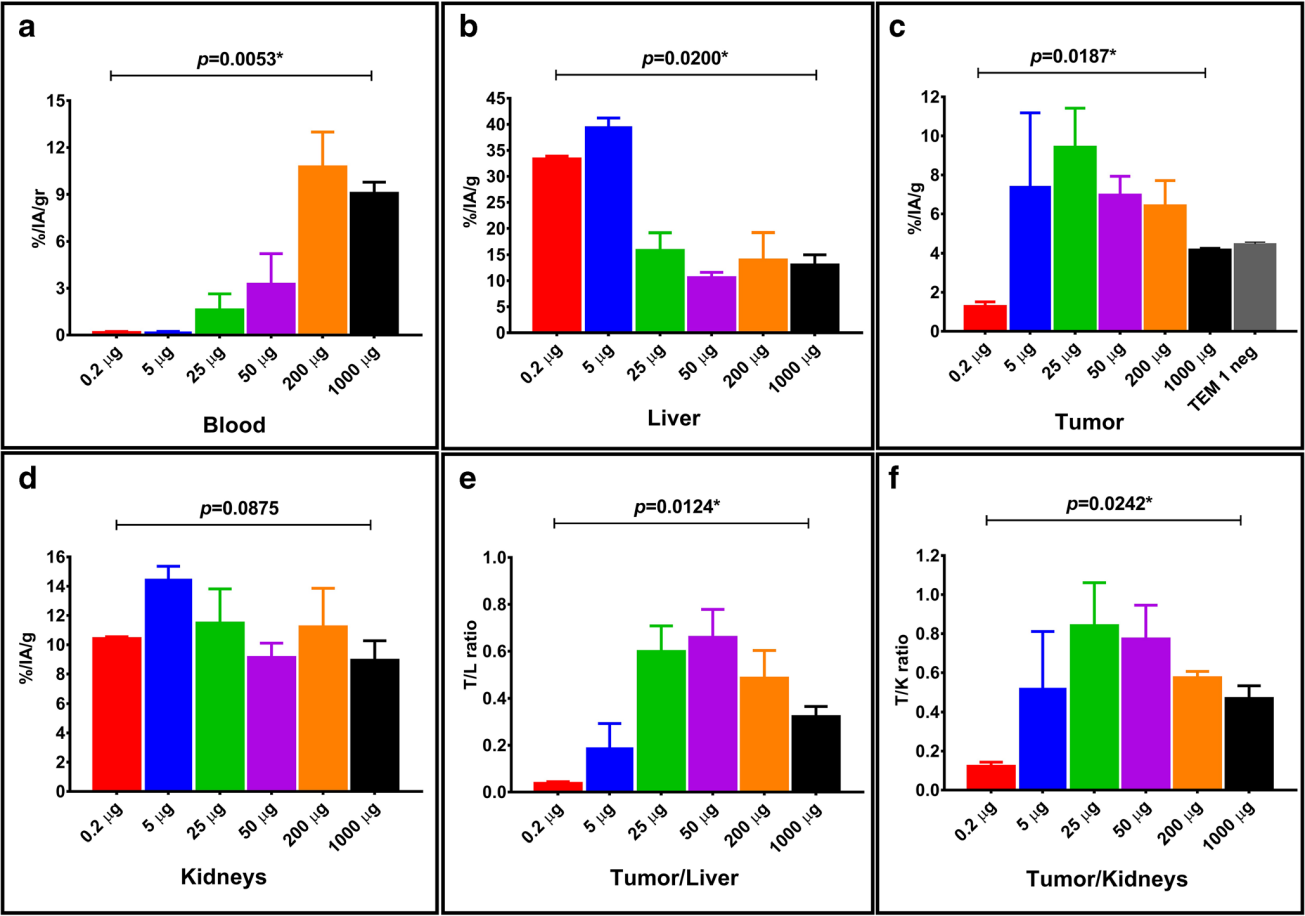
Box-plot panels detailing the biodistribution results of the dose-escalation study in RD-ES tumor-bearing mice. Upper panels show variations of ^111^In-CHX-DTPA-scFv78-Fc uptake in blood (panel **a**), liver (panel **b**) and tumor (panel **c**), respectively. Lower panels show variations of kidney uptake (panel **d**), tumor-to-liver (panel **e**) and tumor-to-kidneys (panel **f**) ratios, respectively. *p* values indicated above each panel were obtained by Kruskall-Wallis test. *p* values < 0.05 are marked with *.

The biodistribution results of the antibody dose-escalation study are shown in Table 1 and Fig. 5. Retention of radioactivity in the blood significantly increased with the total amount of antibody injected (Fig. 5a). On the contrary, splenic uptake was progressively reduced by increasing amounts of antibody injected (*p* = 0.0042, Kruskall–Wallis test) (Table 1). Radiopharmaceutical uptake in the liver dropped significantly between 5 and 25 μg of total antibody injected, being relatively stable thereafter (Fig. 5b).

**Table 1:** Results of the antibody dose-escalation study in RD-ES tumor-bearing mice, sacrificed 24 h after radiopharmaceutical injection. Organ uptake values (mean %IA/g ± SD) are shown for different amounts of total antibody injected (range: 0.2–1000 μg)

Source organ (%IA/g)	0.2 μg AB		5 μg AB		25 μg AB		50 μg AB		200 μg AB		1000 μg AB	
	Mean	SD	Mean	SD	Mean	SD	Mean	SD	Mean	SD	Mean	SD
Blood	0.189	0.041	0.204	0.032	1.634	1.004	3.274	1.939	10.777	2.219	9.092	0.688
Liver	33.404	0.492	39.580	1.646	15.905	3.289	10.656	0.955	14.034	5.203	13.078	1.915
Spleen	114.131	27.447	125.320	2.422	32.636	4.632	23.480	7.554	10.944	0.988	5.781	0.794
Heart	1.508	0.391	2.844	0.183	1.608	0.307	1.669	0.479	3.853	1.017	3.409	0.084
Kidneys	10.449	0.100	14.499	0.867	11.496	2.318	9.157	0.955	11.254	2.609	8.966	1.310
Lungs	3.392	0.452	4.849	0.296	5.290	2.724	3.739	0.779	6.681	0.557	4.884	0.703
Uterus and ovaries	2.155	0.618	3.604	1.359	5.587	2.015	5.716	1.497	6.966	1.869	5.813	1.598
Stomach	0.818	0.631	0.861	0.379	1.161	0.438	0.853	0.148	1.230	0.274	0.934	0.191
Pancreas	0.206	0.115	0.399	0.000	1.423	0.194	1.193	0.200	1.781	0.465	1.257	0.322
Small intestine	1.387	0.082	4.477	1.078	5.349	0.639	3.610	0.359	2.239	0.285	1.589	0.418
Colon	0.791	0.007	2.976	0.374	2.932	0.602	2.909	0.680	2.762	0.572	2.665	1.469
Muscle	0.198	0.019	0.203	0.025	0.541	0.172	0.628	0.225	0.980	0.540	0.994	0.088
Tumor	1.288	0.221	7.423	3.760	9.436	1.986	6.979	0.949	6.435	1.275	4.186	0.067

Tumor uptake was maximum at 25 μg and reduced progressively with larger amounts of antibody injected. Tumor uptake at 1 mg of total antibody injected was similar to the uptake obtained in HT-1080, endosialin/TEM-1-negative tumors injected with 25 μg of total antibody (average %IA/g = 4.18 ± 0.06 *vs.* 4.47 ± 0.09, respectively), indicating the specificity of [^111^In]CHX-DTPA-scFv78-Fc uptake for endosialin/TEM1 (Fig. 5c).

Uptake in the kidneys did not vary significantly with different amounts of antibody injected (Fig. 5d). Tumor-to-liver and tumor-to-kidneys ratios peaked at 50 μg (average ratio = 0.66 ± 0.1) and 25 μg (average ratio = 0.84 ± 0.22) of total antibody injected, respectively, although the differences between 25 µg and 50 µg were not significant (both *p* > 0.3, Mann–Whitney *U* test) (Fig. 5e and f).

**Fig. 6:**
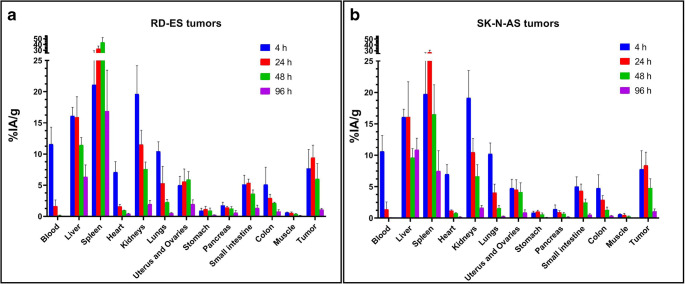
Full biodistribution results of [^111^In]CHX-DTPA-scFv78-Fc in RD-ES (panel **a**) and SK-N-AS (panel **b**) tumor-bearing mice. Mice were sacrificed, and their organs harvested and counted 4, 24, 48 and 96 h after injection (3–5 mice per group). Data are reported as % injected activity/g of tissue (%IA/g). Decay correction was not applied. The data reported in panel **a** were already used to generate the biological organ kinetic of [^111^In]CHX-DTPA-scFv78-Fc shown in [34] by applying radionuclide physical decay correction.

The total antibody doses of 25 and 50 μg showed the most favorable profile in terms of tumor and non-tumor tissue uptake. Eventually, 25 μg was chosen for full biodistribution experiments due to significantly higher absolute tumor uptake compared with 50 μg (*p* = 0.0317, Mann-Whitney *U* test). Results of full biodistribution experiments in RD-ES and SK-N-AS tumor-bearing mice are shown in Fig. 6 (see also Supplementary Table 1 for detailed results). Biodistribution in normal organs and subcutaneous tumors was similar for both tumor models. The only relevant difference was found for the spleen; radiopharmaceutical uptake in the spleen was in continuous uptake between 4 and 48 h in RD-ES-bearing mice, whereas it washed-out after 24 h in SK-N-AS-bearing mice. Liver and tumor uptake peaked at 24 h in both tumor models and showed a washout thereafter. Radioactivity clearance from the liver was more evident for RD-ES- than for SK-N-AS-bearing animals. Uptake in female reproductive organs washed-out after 48 h in both models. In all other organs but the small intestine, the radioactivity washed out already after 4 h.

**Fig. 7:**
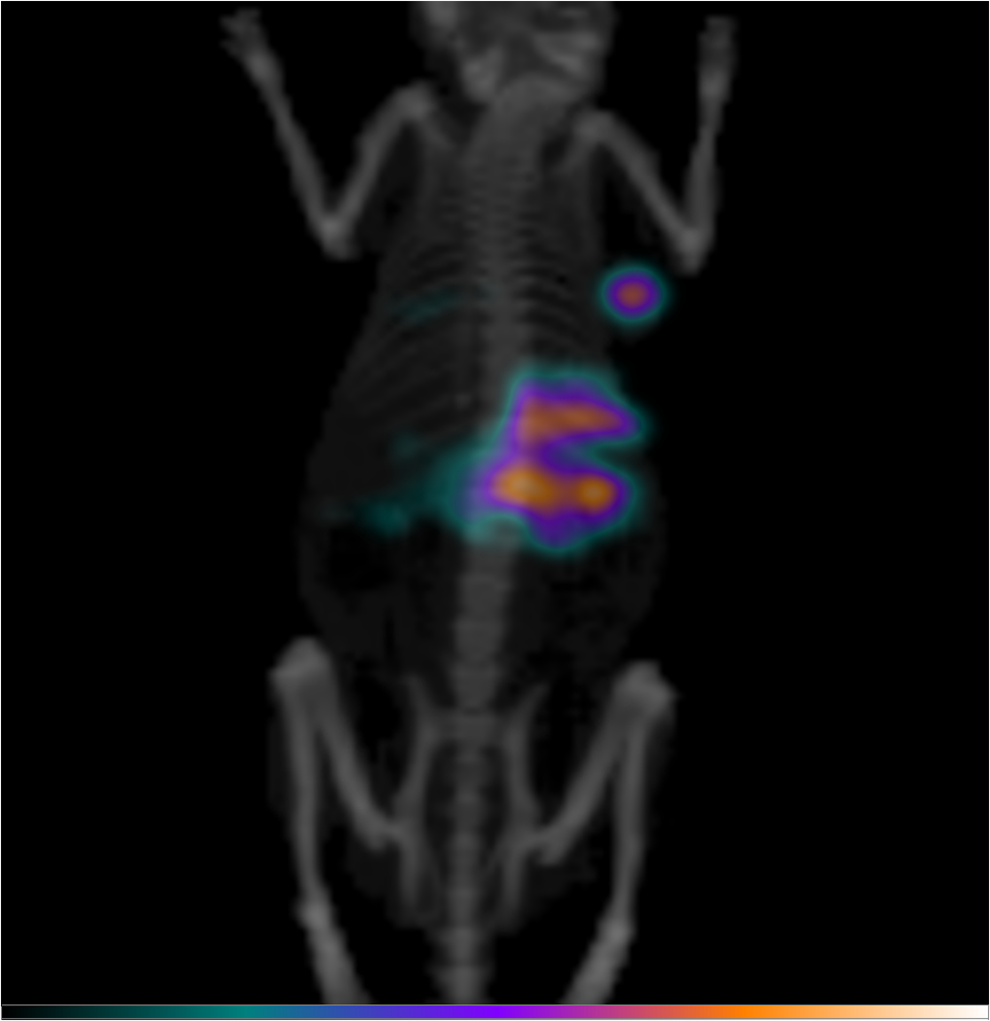
Maximum intensity projection of a SPECT/CT acquired 24 h after the i.v. injection of 5 MBq [^111^In]CHX-DTPA-scFv78-Fc in a RD-ES tumor-bearing mouse (25 μg of total antibody injected). Increased radiopharmaceutical uptake is seen in the right flank, corresponding to the subcutaneous RD-ES tumor. Liver uptake is also observed. The image was acquired using a PET/SPECT/CT device (Albira, Bruker), equipped with a dual-headed SPECT/CT mounting CsI(Tl) scintillation crystals and a single pinhole collimator (FOV80 [44]). The SPECT/CT acquisition consisted of 60 SPECT projections (45 s/projection) and a 45 kV, 200 mA CT. The mouse was anesthetized with isoflurane (2 % in 1 l/min medical air) and warmed on a heating pad during the scan.

A single-photon emission computed tomography (SPECT/CT) image of a RD-ES tumor-bearing mouse acquired after the injection of [^111^In]CHX-DTPA-scFv78-Fc is shown in Fig. 7.

### Dosimetry

Experimentally derived TIACs_m_ and TIACs_m_ corrected for the contribution of tumor sink (TIACs_m,correct_) are reported in Supplementary Table 2. Extrapolated TIACs_h_ for adult male and female models are reported in Supplementary Table 3. Extrapolated absorbed doses for adult male, female and reference person deriving from the injection of [^111^In]CHX-DTPA-scFv78-Fc are shown in Table 2. The organ receiving the highest absorbed dose from a theoretical injection of [^111^In]CHX-DTPA-scFv78-Fc to the reference person would be the spleen (0.876 mGy/MBq), followed by the liver (0.570 mGy/MBq) and the kidneys (0.298 mGy/MBq). The total body dose and the effective dose would be 0.058 mGy/MBq and 0.116 mSv/MBq, respectively.

**Table 2:** Human extrapolated dosimetry of [^111^In]CHX-DTPA-scFv78-Fc. Average absorbed dose estimates (mGy/Mq) are reported for adult male, female and reference person. In addition, theoretical upper and lower dose ranges are shown. These estimates come from mouse TIACs calculated from source organ time-activity curves obtained by fitting nA + SD and nA-SD values, respectively. The effective dose (mSv/MBq) is provided for the gender-averaged model, according to the ICRP 103 methodology [39]. *N/A* not applicable

Target organ	Absorbed dose (mGy/Mq) Male			Absorbed dose (mGy/Mq) Female			Absorbed dose (mGy/Mq) Gender averaged		
	Average	Lower range	Upper range	Average	Lower range	Upper range	Average	Lower range	Upper range
Adrenals	2.50E−01	1.85E−01	3.15E−01	3.80E−01	2.76E−01	4.82E−01	3.15E−01	2.30E−01	3.98E−01
Brain	2.39E−02	1.66E−02	3.07E−02	2.47E−02	1.70E−02	3.15E−02	2.43E−02	1.68E−02	3.11E−02
Breasts	N/A	N/A	N/A	4.09E−02	2.83E−02	5.25E−02	4.09E−02	2.83E−02	5.25E−02
Esophagus	1.02E−01	7.08E−02	1.31E−01	1.18E−01	8.23E−02	1.51E−01	1.10E−01	7.65E−02	1.41E−01
Eyes	2.40E−02	1.66E−02	3.07E−02	2.49E−02	1.70E−02	3.17E−02	2.44E−02	1.68E−02	3.12E−02
Gallbladder wall	2.51E−01	1.86E−01	3.18E−01	1.89E−01	1.41E−01	2.38E−01	2.20E−01	1.63E−01	2.78E−01
Left colon	1.03E−01	7.84E−02	1.27E−01	1.11E−01	8.32E−02	1.38E−01	1.07E−01	8.08E−02	1.33E−01
Small Intestine	1.37E−01	1.07E−01	1.66E−01	1.72E−01	1.34E−01	2.07E−01	1.55E−01	1.21E−01	1.87E−01
Stomach wall	1.16E−01	8.12E−02	1.62E−01	1.45E−01	1.03E−01	2.02E−01	1.30E−01	9.21E−02	1.82E−01
Right colon	1.16E−01	8.92E−02	1.43E−01	1.10E−01	8.57E−02	1.35E−01	1.13E−01	8.74E−02	1.39E−01
Rectum	6.08E−02	4.77E−02	7.37E−02	7.13E−02	5.52E−02	8.68E−02	6.61E−02	5.14E−02	8.03E−02
Heart wall	1.30E−01	7.66E−02	1.63E−01	1.25E−01	6.88E−02	1.56E−01	1.28E−01	7.27E−02	1.60E−01
Kidneys	2.68E−01	2.07E−01	3.26E−01	3.28E−01	2.53E−01	3.99E−01	2.98E−01	2.30E−01	3.63E−01
Liver	5.25E−01	3.89E−01	6.66E−01	6.15E−01	4.56E−01	7.80E−01	5.70E−01	4.22E−01	7.23E−01
Lungs	1.20E−01	7.99E−02	1.59E−01	1.37E−01	9.02E−02	1.80E−01	1.29E−01	8.51E−02	1.70E−01
Ovaries	N/A	N/A	N/A	1.42E−01	1.00E−01	1.81E−01	1.42E−01	1.00E−01	1.81E−01
Pancreas	1.48E−01	1.06E−01	1.94E−01	2.16E−01	1.56E−01	2.79E−01	1.82E−01	1.31E−01	2.36E−01
Prostate	4.37E−02	3.15E−02	5.51E−02	N/A	N/A	N/A	4.37E−02	3.15E−02	5.51E−02
Salivary glands	3.00E−02	2.07E−02	3.85E−02	2.74E−02	1.88E−02	3.50E−02	2.87E−02	1.98E−02	3.68E−02
Red marrow	6.38E−02	4.33E−02	8.03E−02	7.07E−02	4.80E−02	8.85E−02	6.73E−02	4.57E−02	8.44E−02
Osteogenic cells	7.31E−02	5.07E−02	9.27E−02	7.84E−02	5.43E−02	9.90E−02	7.58E−02	5.25E−02	9.59E−02
Spleen	8.00E−01	5.67E−01	1.02E+00	9.53E−01	6.80E−01	1.22E+00	8.76E−01	6.24E−01	1.12E+00
Testes	2.56E−02	1.79E−02	3.28E−02	N/A	N/A	N/A	2.56E−02	1.79E−02	3.28E−02
Thymus	6.33E−02	4.16E−02	8.11E−02	7.24E−02	4.75E−02	9.33E−02	6.78E−02	4.45E−02	8.72E−02
Thyroid	4.37E−02	2.97E−02	5.67E−02	4.16E−02	2.80E−02	5.37E−02	4.26E−02	2.89E−02	5.52E−02
Urinary bladder wall	3.82E−02	2.72E−02	4.84E−02	4.57E−02	3.29E−02	5.75E−02	4.20E−02	3.00E−02	5.30E−02
Uterus	N/A	N/A	N/A	1.65E−01	1.17E−01	2.10E−01	1.65E−01	1.17E−01	2.10E−01
Total body	4.65E−02	3.29E−02	5.91E−02	6.99E−02	5.00E−02	8.86E−02	5.82E−02	4.15E−02	7.39E−02
Effective dose (mSv/MBq)							1.16E−01	8.25E−02	1.49E−01

## Discussion

At present, it is widely recognized that the tumor microenvironment plays a pivotal role in tumor progression and therapeutic response. Tumor stroma and neovasculature are essential constituents of tumor microenvironment and represent the target of several novel anticancer treatments [45]. Endosialin/TEM1 has emerged as an attractive surface molecule for molecular imaging and therapeutic applications, being expressed by the neovasculature and by the stroma of a wide range of tumors [23]. Among all the cells composing the tumor stroma, cancer-associated fibroblasts deserve a special mention since they represent one of the most abundant stromal sub-populations, which play critical roles in shaping the tumor ecosystem. Despite their phenotypical and functional heterogeneity, these cells have been widely described to actively participate in all steps of tumor evolution: providing physical support to the malignant cells, modulating their tumorigenic properties and regulating the tumor-immune interactions [46].

Some tumor types, like sarcomas and neuroblastoma, express endosialin/TEM1 on both tumor vasculature and tumor cells. For these tumors, targeting endosialin/TEM1 could represent not only a strategy to interfere with the tumor microenvironment by disrupting the tumor neovasculature but it might also have a direct anticancer effect [11, 20, 22]. Endosialin/TEM1-targeting antibodies are already being tested in clinical trials [23–27], and the development of a specific diagnostic probe might help to qualify patients for receiving these types of treatments.

The availability of human tumorigenic cell lines expressing endosialin/TEM1 makes the assessment of endosialin-targeting probes possible with common human xenograft tumor preclinical models.

In the present study, a fully human anti-endosialin/TEM1 scFv derivative fused to an immunoglobulin Fc was radiolabeled with indium-111 and evaluated preclinically.

The radioimmunoconjugate was tested in immunodeficient mice bearing human Ewing’s sarcoma RD-ES and neuroblastoma SK-N-AS tumors. The biodistribution results of [^111^In]CHX-DTPA-scFv78-Fc in RD-ES tumor-bearing mice were already used in a previous publication in order to extrapolate mouse dosimetry data to be compared with direct image-based [^152^Tb]CHX-DTPA-scFv78-Fc mouse dosimetry [34]. The present work extends previously published results by reporting on the full synthesis process and *in vitro* assessment of [^111^In]CHX-DTPA-scFv78-Fc, by evaluating an additional tumor model of human neuroblastoma and by providing human dose extrapolations from mice biodistribution data.

The radiolabeled product [^111^In]CHX-DTPA-scFv78-Fc was obtained with a radiochemical purity > 98 % after one hour incubation at 42 degrees and ultrafiltration and proved to be stable after 96 h at 37 °C in human serum. In addition, the immunoreactive fraction *r* extrapolated to infinite antigen excess was > 70 %, and *K*_D_ was in the same range of values reported for radio-iodinated scFv fusion derivatives [33].

Positivity of both tumor models for endosialin/TEM1 staining was confirmed by FACS on cultured cells and by immunohistochemistry on subcutaneously grown tumors. Biodistribution data acquired in tumor-bearing mice confirmed fast blood clearance of the [^111^In]CHX-DTPA-scFv78-Fc and positive tumor targeting in both xenograft models. The radiopharmaceutical off-target uptake was predominantly abdominal, with the liver and the spleen showing the highest uptake values. This biodistribution pattern is not due to specific TEM1 expression by abdominal organs [5, 32], but it is common to all antibodies bearing a functional Fc region [47]. We observed some differences of uptake profiles in the liver and the spleen between the two xenograft tumor models. This variability might have been caused by the heterogeneity of the number of chelators per molecule among the different batches of radiolabeled product that were used for the experiments. In fact, it has been shown that the number of chelators has an impact on the biodistribution of the radioimmunoconjugates, particularly in abdominal organs [48, 49].

The full biodistribution studies were preceded by a pharmaceutical dose-escalation preliminary experiment based on the simultaneous administration of increasing amounts of unlabeled scFv78-Fc at the time of the injection of the radioimmunoconjugate. Our results showed an optimized biodistribution at 25 μg (1 mg/kg) of total antibody injected, and progressive saturation of tumor targeting with increasing total antibody dose, indicating the specificity of tumor uptake. The optimal total antibody amount of 1 mg/kg injected in mice would correspond to 5.7 mg of total antibody injected in a human subject weighing 70 kg [50]. However, it is possible that the biodistribution of [^111^In]CHX-DTPA-scFv78-Fc could be further improved by predosing rather than by a simultaneous injection of unlabeled antibody [51].

The cross-species reactivity of scFv78-Fc is a significant potential advantage for its clinical translation. In fact, a human antibody cross-reactive with a mouse antigen behaves more physiologically in a mouse xenograft model, and the resulting biodistribution data are more informative than those obtained with non-cross-reactive compounds [52]. Given the growing interest in generating internal dosimetry estimates, particularly for newer radiopharmaceuticals [53], we used mouse biodistribution data to extrapolate radiation absorbed doses to humans for a theoretical radiopharmaceutical administration having a diagnostic purpose. Human dosimetry extrapolations confirmed the spleen, the liver and the kidneys to be the organs potentially receiving the highest doses from the injection of [^111^In]CHX-DTPA-scFv78-Fc. Interestingly, these dose extrapolations compare well with the absorbed dose calculations of the MIRD Committee for [^111^In]Ibritumomab-tiuxetan, that is the diagnostic counterpart of [^90^Y]Ibritumomab-tiuxetan (Zevalin®) [54]. Zevalin® was the only antibody labeled with radiometals approved for human for the treatment of recurrent/refractory non-Hodgkin’s lymphomas [55]. In particular, according to our extrapolations, [^111^In]CHX-DTPA-scFv78-Fc would deliver a 22 % higher absorbed dose to the liver (0.570 *vs.* 0.467 mGy/MBq) and a 5 % lower dose to the kidneys (0.298 *vs.* 0.315 mGy/MBq) compared with [^111^In]Ibritumomab-tiuxetan [54]. A larger discrepancy of 89 % would be observed for the spleen (0.876 *vs.* 0.464 mGy/MBq for [^111^In]CHX-DTPA-scFv78-Fc and [^111^In]Ibritumomab-tiuxetan, respectively). It should be noted, however, that our dose estimations for the spleen are very conservative since our extrapolations are based on the biodistribution data obtained from RD-ES tumor-bearing mice, which showed a higher retention of radioactivity in this organ compared with SK-N-AS tumor-bearing mice (Fig. 6). Nevertheless, the total-body absorbed dose would be 53 % lower for [^111^In]CHX-DTPA-scFv78-Fc compared with [^111^In]Ibritumomab-tiuxetan (0.058 *vs.* 0.124 mGy/MBq) [54].

Some considerations deserve to be made regarding the methodology used for dose extrapolations from mice to humans. There are two popular methods for extrapolating human dosimetry from small animal biodistribution data [56]. In the method adopted here, which is also supported by our federal guidelines [41], the source organ TIACs are rescaled by taking into account the relative contribution of single organ masses to the total body weight in both species [57]. Another method does not consider rescaling the TIACs, rather it applies the TIACs measured on mice directly on human organ masses (we call it “direct method” thereafter). In order to explore possible discrepancies between these two methods, we performed dosimetry extrapolations with this latter method as well. The highest discrepancies would be obtained for those organs whose relative mass is most different between mouse and humans, such as the female reproductive organs. In fact, the direct method would give absorbed doses 5.6 (0.927 *vs.* 0.165 mGy/MBq) and 13.2 (1.88 *vs.* 0.142 mGy/MBq) times higher for uterus and ovaries, respectively, compared with the method we adopted. Smaller, but still relevant, overestimations of 65 and 95 % would be obtained with the direct method for other important organs, such as the liver (0.921 *vs.* 0.570 mGy/MBq) and the kidneys (0.580 *vs.* 0.298 mGy/MBq), respectively (full data not shown). Using a different radiopharmaceutical, some authors have found a better agreement between the two methodologies for dose extrapolations [58]; however, the discrepancies between the two methods are tracer-specific, which makes hard to draw general conclusions. It remains to be determined which methodology would better predict the absorbed doses in real patients. In a previous work on the integrin-targeting peptide [^68^Ga]NODAGA-RGDyK, we found a quite good agreement between absorbed doses extrapolated from mice using the direct method and real patients’ data [59]. Additional procedures for interspecies dose extrapolations exist, which take into account also a time scaling [60], but they have not been considered here. Furthermore, since our biodistribution studies were conducted on tumor-bearing mice, we have introduced an additional correction of the mouse TIAC to take into account the contribution of the tumor antigenic sink. More specifically, the mouse tumor TIAC was redistributed into mouse source-organ TIACs proportionally to their contribution to the whole-body TIAC. In our opinion, this correction would allow a more meaningful dose extrapolation to the reference human models, where the presence of tumor is not considered. Nevertheless, to the best of our knowledge, this method is original and warrants further validation.

In summary, we have shown that [^111^In]CHX-DTPA-scFv78-Fc specifically binds to the TEM1 antigen *in vitro* and confirmed tumor targeting in two mouse xenograft models of endosialin/TEM1-expressing human tumors. Finally, our dosimetry extrapolations to humans are in the range of other monoclonal antibodies radiolabeled with indium-111. Our results suggest that [^111^In]CHX-DTPA-scFv78-Fc deserves further consideration for a possible clinical translation as a theranostic probe.

## Electronic Supplementary Material

ESM 1 (DOCX 35 kb)
