# Supplementary material for: Preclinical Evaluation and Dosimetry of [111In]CHX-DTPA-scFv78-Fc Targeting Endosialin/Tumor Endothelial Marker 1 (TEM1)
Source: Mol Imaging Biol. 2020 Jan 28;22(4):979–91. doi: 10.1007/s11307-020-01479-8 (PMC7343747; doi:10.1007/s11307-020-01479-8)
Supplement: Supplementary file 1 — (DOCX 35 kb) [file 11307_2020_1479_MOESM1_ESM.docx]

**Electronic Supplementary Material**

**Article Title**: Preclinical evaluation and dosimetry of [^111^In]CHX-DTPA-scFv78-Fc targeting endosialin/tumor endothelial marker 1 (TEM1)

**Journal**: Molecular Imaging and Biology

**Authors**: Francesco Cicone^1,2^, Thibaut Denoël^1^, Silvano Gnesin^3^, Nicolo Riggi^4^, Melita Irving^5^, Gopinadh Jakka^5^, Niklaus Schaefer^1^, David Viertl^1^, George Coukos^5^, John O. Prior^1^.

**Affiliations**:

^1^Department of Nuclear Medicine and Molecular Imaging, Lausanne University Hospital and University of Lausanne, Lausanne, Switzerland

^2^Unit of Nuclear Medicine, Department of Experimental and Clinical Medicine, “Magna Graecia” University of Catanzaro, Catanzaro, Italy

^3^Institute of Radiation Physics, Lausanne University Hospital and University of Lausanne, Lausanne, Switzerland

^4^Experimental Pathology Service, Institute of Pathology, Lausanne University Hospital and University of Lausanne, Lausanne, Switzerland.

^5^Ludwig Institute for Cancer Research, Lausanne Branch, Department of Oncology, University of Lausanne, CH-1066 Epalinges, Switzerland

**Corresponding Author:**

Francesco Cicone, MD, PhD. (https://orcid.org/0000-0003-4664-1965)

Department of Nuclear Medicine and Molecular Imaging,

Lausanne University Hospital, Rue du Bugnon 46, CH-1011 Lausanne (CH).

Email: [f.cicone@iol.it](mailto:f.cicone@iol.it). TEL: +41 (0)21 3140388

| **RD-ES** | **4 h** | | **24 h** | | **48 h** | | **96 h** | |
| --- | --- | --- | --- | --- | --- | --- | --- | --- |
|  | **Mean** | **SD** | **Mean** | **SD** | **Mean** | **SD** | **Mean** | **SD** |
| Blood | 11.579 | 2.727 | 1.634 | 1.004 | 0.156 | 0.050 | 0.020 | 0.003 |
| Liver | 16.127 | 1.366 | 15.905 | 3.289 | 11.442 | 1.203 | 6.351 | 1.901 |
| Spleen | 21.118 | 8.011 | 32.636 | 4.632 | 43.714 | 8.171 | 16.890 | 6.567 |
| Heart | 7.087 | 1.685 | 1.608 | 0.307 | 0.939 | 0.071 | 0.410 | 0.094 |
| Kidneys | 19.612 | 4.551 | 11.496 | 2.318 | 7.570 | 1.152 | 1.946 | 0.596 |
| Lungs | 10.423 | 1.539 | 5.290 | 2.724 | 2.284 | 0.426 | 0.504 | 0.114 |
| Uterus and Ovaries | 4.997 | 1.425 | 5.587 | 2.015 | 5.926 | 1.240 | 1.962 | 0.681 |
| Stomach | 0.908 | 0.401 | 1.161 | 0.438 | 0.905 | 0.430 | 0.237 | 0.031 |
| Pancreas | 1.747 | 0.523 | 1.423 | 0.194 | 1.253 | 0.311 | 0.564 | 0.360 |
| Small intestine | 5.131 | 1.461 | 5.349 | 0.639 | 3.633 | 0.604 | 1.352 | 0.456 |
| Colon | 5.112 | 2.767 | 2.932 | 0.602 | 2.137 | 0.131 | 0.768 | 0.298 |
| Muscle | 0.602 | 0.096 | 0.541 | 0.172 | 0.371 | 0.087 | 0.122 | 0.031 |
| Tumor | 7.689 | 3.044 | 9.436 | 1.986 | 6.022 | 2.461 | 1.113 | 0.166 |
| **SK-N-AS** |  | | | | | | | |
|  |  |  |  |  |  |  |  |  |
| Blood | 10.639 | 2.493 | 1.402 | 1.169 | 0.073 | 0.024 | 0.015 | 0.002 |
| Liver | 16.098 | 1.245 | 16.103 | 5.590 | 9.627 | 1.433 | 10.893 | 1.825 |
| Spleen | 19.768 | 6.855 | 27.194 | 3.738 | 16.500 | 4.711 | 7.494 | 3.233 |
| Heart | 6.981 | 1.532 | 1.168 | 0.145 | 0.768 | 0.096 | 0.181 | 0.040 |
| Kidneys | 19.144 | 4.339 | 10.469 | 2.200 | 6.651 | 1.839 | 1.650 | 0.351 |
| Lungs | 10.219 | 1.736 | 4.057 | 1.350 | 1.583 | 0.398 | 0.289 | 0.061 |
| Uterus and Ovaries | 4.777 | 1.396 | 4.500 | 1.602 | 4.124 | 1.497 | 0.903 | 0.402 |
| Stomach | 0.856 | 0.257 | 1.043 | 0.210 | 0.568 | 0.251 | 0.099 | 0.031 |
| Pancreas | 1.444 | 0.657 | 0.910 | 0.282 | 0.689 | 0.203 | 0.171 | 0.045 |
| Small intestine | 5.031 | 1.541 | 4.342 | 1.076 | 2.472 | 0.550 | 0.550 | 0.140 |
| Colon | 4.762 | 2.133 | 2.923 | 0.638 | 1.294 | 0.457 | 0.359 | 0.068 |
| Muscle | 0.592 | 0.085 | 0.482 | 0.261 | 0.252 | 0.096 | 0.066 | 0.021 |
| Tumor | 7.779 | 2.947 | 8.387 | 2.103 | 4.789 | 1.465 | 1.084 | 0.372 |

**Supplementary Table 1**. Full biostribution results of [^111^In]CHX-DTPA-scFv78-Fc in RD-ES and SK-N-AS tumor-bearing mice. For each source organ, the table shows uptake values (mean %IA/g ± SD) at different time points after radiopharmaceutical injection.

|  | **TIACs_m_ (MBq.h/MBq)** | | | **TIACs_m,corrected_ (MBq.h/MBq)** | | |
| --- | --- | --- | --- | --- | --- | --- |
| **Source organ** | **Average** | **Lower range** | **Upper range** | **Average** | **Lower range** | **Upper range** |
| Blood (total) | 2.55E+00 | 1.65E+00 | 3.45E+00 | 2.94E+00 | 1.65E+00 | 3.45E+00 |
| Red marrow | / | / | / | / | / | / |
| Heart carvity | / | / | / | / | / | / |
| Liver | 1.82E+01 | 1.37E+01 | 2.32E+01 | 2.11E+01 | 1.57E+01 | 2.67E+01 |
| Spleen | 1.80E+00 | 1.29E+00 | 2.30E+00 | 2.08E+00 | 1.48E+00 | 2.65E+00 |
| Heart wall | 1.37E-01 | 5.10E-03 | 1.64E-01 | 1.59E-01 | 5.83E-03 | 1.88E-01 |
| Kidneys | 2.32E+00 | 1.89E+00 | 2.75E+00 | 2.69E+00 | 2.16E+00 | 3.17E+00 |
| Lungs | 6.01E-01 | 3.65E-01 | 8.35E-01 | 6.97E-01 | 4.17E-01 | 9.62E-01 |
| Uterus | 1.40E+00 | 9.87E-01 | 1.83E+00 | 1.63E+00 | 1.13E+00 | 2.10E+00 |
| Ovaries | 1.93E-01 | 1.36E-01 | 2.51E-01 | 2.24E-01 | 1.55E-01 | 2.89E-01 |
| Stomach | 5.74E-01 | 3.76E-01 | 2.08E+00 | 6.65E-01 | 4.30E-01 | 2.39E+00 |
| Pancreas | 1.76E-01 | 1.15E-01 | 2.51E-01 | 2.04E-01 | 1.31E-01 | 2.89E-01 |
| Small intestine | 6.44E+00 | 5.39E+00 | 7.50E+00 | 7.47E+00 | 6.16E+00 | 8.64E+00 |
| Left colon | 4.84E-01 | 1.35E+00 | 1.87E+00 | 5.61E-01 | 5.31E-01 | 6.18E-01 |
| Right colon | 9.67E-01 | 2.69E+00 | 3.75E+00 | 1.12E+00 | 1.06E+00 | 1.24E+00 |
| Rectum | 4.84E-01 | 1.35E+00 | 1.87E+00 | 5.61E-01 | 5.31E-01 | 6.18E-01 |
| Remainder | 8.78E+00 | 6.16E+00 | 1.13E+01 | 1.02E+01 | 7.04E+00 | 1.30E+01 |
| Tumor | 6.85E+00 | 4.87E+00 | 8.84E+00 | / | / | / |

**Supplementary Table 2**. Mouse time-integrated activity coefficients (TIACs_m_). TIACs_m_ were obtained from experimental biodistribution data on RD-ES-bearing mice. Corrected mouse TIACs (TIACs_m,correct_**)** were derived from TIACs_m_ under the assumptions detailed in the manuscript.

|  | **TIACs_h_ male (MBq.h/MBq)** | | | **TIACs_h_ female (MBq.h/MBq)** | | |
| --- | --- | --- | --- | --- | --- | --- |
| **Source organ** | **Average** | **Lower range** | **Upper range** | **Average** | **Lower range** | **Upper range** |
| Blood | 3.38E+00 | 1.90E+00 | 3.97E+00 | 2.99E+00 | 1.69E+00 | 3.52E+00 |
| Red marrow | 3.51E-01 | 1.98E-01 | 4.13E-01 | 3.10E-01 | 1.75E-01 | 3.65E-01 |
| Heart carvity | 8.06E-01 | 4.54E-01 | 9.48E-01 | 7.54E-01 | 4.25E-01 | 8.87E-01 |
| Liver | 1.34E+01 | 9.97E+00 | 1.70E+01 | 1.27E+01 | 9.44E+00 | 1.61E+01 |
| Spleen | 2.74E+00 | 1.94E+00 | 3.48E+00 | 2.88E+00 | 2.05E+00 | 3.67E+00 |
| Heart wall | 1.81E-01 | 6.66E-03 | 2.15E-01 | 1.67E-01 | 6.14E-03 | 1.98E-01 |
| Kidneys | 1.11E+00 | 8.96E-01 | 1.31E+00 | 1.21E+00 | 9.70E-01 | 1.42E+00 |
| Lungs | 1.66E+00 | 9.97E-01 | 2.30E+00 | 1.60E+00 | 9.60E-01 | 2.21E+00 |
| Uterus | N/A | N/A | N/A | 2.45E-01 | 1.70E-01 | 3.16E-01 |
| Ovaries | N/A | N/A | N/A | 3.37E-02 | 2.33E-02 | 4.35E-02 |
| Stomach | 5.82E-02 | 3.77E-02 | 2.09E-01 | 6.61E-02 | 4.28E-02 | 2.38E-01 |
| Pancreas | 9.49E-02 | 6.09E-02 | 1.34E-01 | 9.90E-02 | 6.35E-02 | 1.40E-01 |
| Small intestine | 1.32E+00 | 1.09E+00 | 1.53E+00 | 1.48E+00 | 1.22E+00 | 1.71E+00 |
| Left colon | 7.56E-02 | 7.16E-02 | 8.33E-02 | 8.58E-02 | 1.68E-01 | 1.96E-01 |
| Right colon | 1.51E-01 | 1.43E-01 | 1.67E-01 | 1.78E-01 | 1.68E-01 | 1.96E-01 |
| Rectum | 7.56E-02 | 7.16E-02 | 8.33E-02 | 8.58E-02 | 8.13E-02 | 9.46E-02 |
| Remainder | 1.58E+01 | 1.10E+01 | 2.03E+01 | 1.29E+01 | 8.93E+00 | 1.65E+01 |

**Supplementary Table 3**. Human time-integrated activity coefficients (TIACs_h_), derived from corrected mouse TIACs (reported in Supplementary Table 2).
